# Case Report: Bullous erythema multiforme induced by omalizumab

**DOI:** 10.3389/fmed.2026.1881122

**Published:** 2026-07-17

**Authors:** Zenan Tang, Yan Zhao

**Affiliations:** Department of Dermatology, Peking University People's Hospital, Beijing, China

**Keywords:** adverse drug reaction, bullous erythema multiforme, case report, chronic spontaneous urticaria, omalizumab

## Abstract

Bullous erythema multiforme (BEM) is an acute immune-mediated mucocutaneous disease, with drug induction being one of its common causes. Omalizumab, a recombinant humanized IgG1 monoclonal antibody targeting free IgE, is widely used in chronic spontaneous urticaria and asthma due to its favorable safety profile, while severe cutaneous bullous adverse reactions induced by it are extremely rare. We report a 36-year-old female patient with a history of bronchial asthma, allergic rhinitis, allergic conjunctivitis, and chronic spontaneous urticaria who developed generalized wheals. After the first subcutaneous injection of omalizumab 300 mg, multiple raised, edematous erythematous papules and plaques, vesicles, and bullae appeared on the trunk, limbs, palms, soles, and oral mucosa 17 days later, accompanied by fever, chills, and fatigue. Laboratory examinations showed elevated eosinophil count and IgE level, and skin biopsy from the left upper limb revealed focal epidermal necrotic keratinocytes, intraepidermal and subepidermal blister formation, and massive eosinophilic infiltration. The patient was diagnosed with omalizumab-induced BEM. Initial treatment with methylprednisolone combined with intravenous immunoglobulin (IVIG) was ineffective. The condition was successfully controlled after increasing the dose of methylprednisolone and adding oral upadacitinib. This case suggests that omalizumab may induce BEM, and combination therapy with high-dose glucocorticoids and JAK inhibitors may be an effective strategy for refractory cases. Clinicians should be alert to such rare adverse reactions when using omalizumab.

## 1 Introduction

Bullous erythema multiforme (BEM) is an acute immune-mediated mucocutaneous disease characterized by typical targetoid lesions accompanied by vesicles or bullae formation. It frequently involves multiple mucosal sites such as the oral cavity, genitalia, and conjunctiva (1). Common triggers include infections (e.g., herpes simplex virus), medications, and malignancies. The most frequently implicated drugs are non-steroidal anti-inflammatory drugs (NSAIDs), sulfonamides, anticonvulsants, and antibiotics. BEM can also occur in association with malignancies, autoimmune diseases, radiation, immunization, and menstruation (2). Because BEM may be triggered by both conventional medications and newer biologic agents, careful assessment of recent drug exposure is essential when typical targetoid and bullous lesions develop.

Omalizumab is a recombinant humanized IgG1 monoclonal antibody that binds to free IgE, thereby blocking the interaction between free IgE and FcεRI receptors on the surface of mast cells and basophils. Omalizumab also inhibits IgE-dependent antigen-presentation and suppresses Th2-mediated inflammatory pathways, potentially preventing IgE-mediated allergic reactions through multiple axes (3). Multiple clinical trials and meta-analyses have demonstrated a favorable safety and tolerability profile for omalizumab, with common adverse reactions including nasopharyngitis, sinusitis, viral upper respiratory tract infection, headache, arthralgia, and cough (4, 5). Nevertheless, rare immune-mediated cutaneous adverse events can occur and may be difficult to recognize because of the generally favorable safety profile of omalizumab. Here we report a case of bullous erythema multiforme induced by omalizumab.

## 2 Case description

The patient was a 36-year-old woman with a history of chronic spontaneous urticaria (CsU). According to a review of her outpatient records from both our hospital and an outside hospital, she had experienced recurrent wheals for more than 1 month before admission and had visited our outpatient clinic several times. She had been treated with oral antihistamines, including mizolastine 10 mg once daily and cetirizine 10 mg every night, but her urticaria remained poorly controlled. Therefore, omalizumab was initiated for antihistamine-refractory CsU. On February 6, 2025, she received her first subcutaneous injection of omalizumab 300 mg, while continuing oral mizolastine 10 mg once daily and cetirizine 10 mg every night. On February 23, 2025, 17 days after the first omalizumab dose, multiple raised, edematous erythematous papules and plaques developed on the trunk and limbs, with vesicles and bullae on the hands and feet. These lesions were accompanied by significant pain and severe pruritus. Concurrently, scattered vesicles were observed on the oral mucosa, accompanied by a sore throat that worsened upon swallowing, as well as fever, chills, and fatigue. Before this episode, the patient denied any medication use other than the treatments prescribed for urticaria.

She had a history of cervical HPV infection, bronchial asthma, allergic rhinitis, allergic conjunctivitis, and chronic spontaneous urticaria.

**Figure 1:**
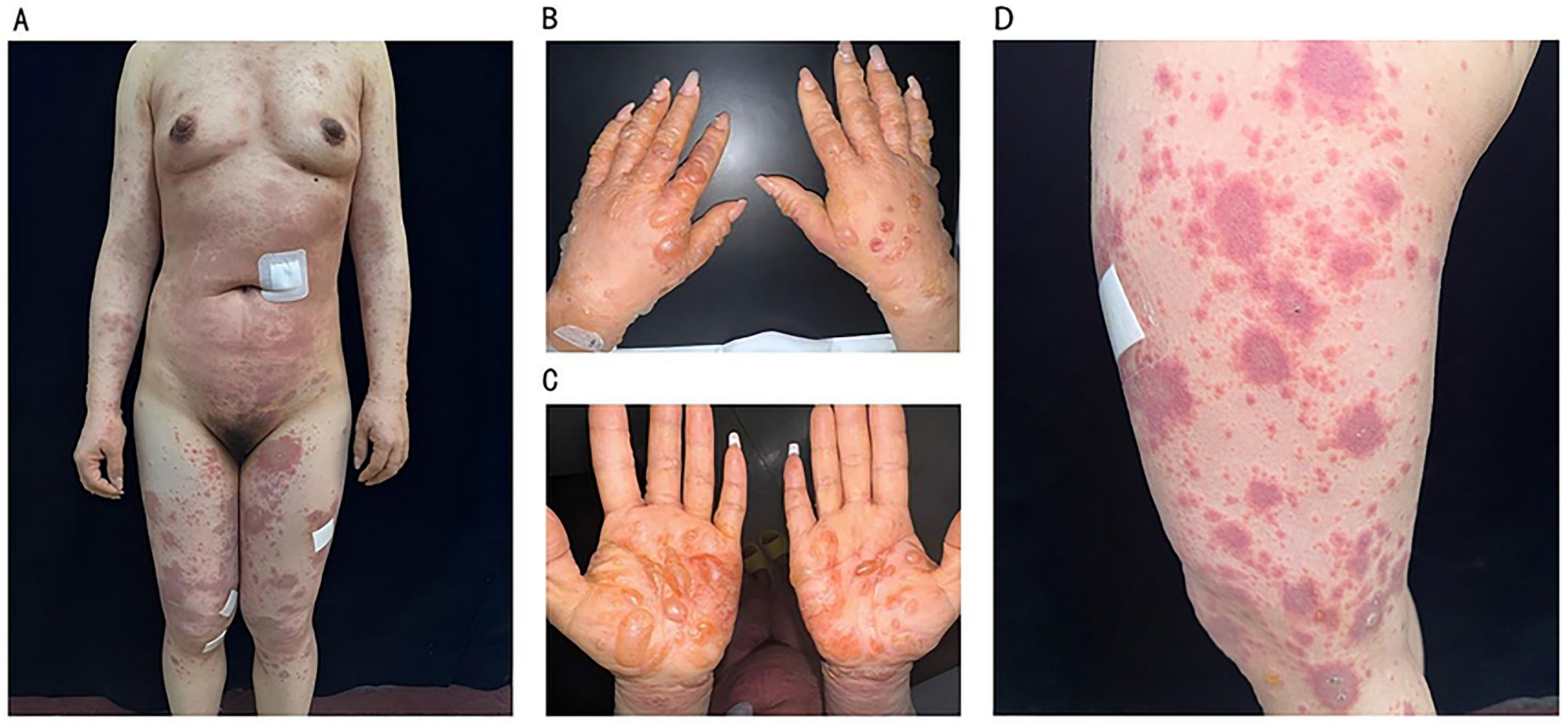
Clinical images before treatment. **(A)** Multiple raised, edematous erythematous papules and plaques with vesicles and bullae diffusely distributed on the trunk and limbs. **(B)** Vesicles and bullae with clear fluid and crusted erosions on the dorsal hands. **(C)** Multiple tense bullae and erosions on the palms. **(D)** Multiple purplish-red targetoid lesions with central clearing on the thigh.

Physical examination revealed numerous raised, edematous, erythematous papules and plaques diffusely distributed on the trunk and limbs, some appearing as purplish-red targetoid lesions. Vesicles and bullae with clear fluid and thick walls were also observed on the palms, wrists, soles, and medial aspects of both feet, some of which had ruptured and crusted. The Nikolsky sign was negative. Scattered small vesicles and erosions were present on the bilateral buccal mucosa. No abnormalities were detected on the conjunctival or genital mucosa (Figure 1).

### 2.1 Diagnostic assessment

**Figure 2:**
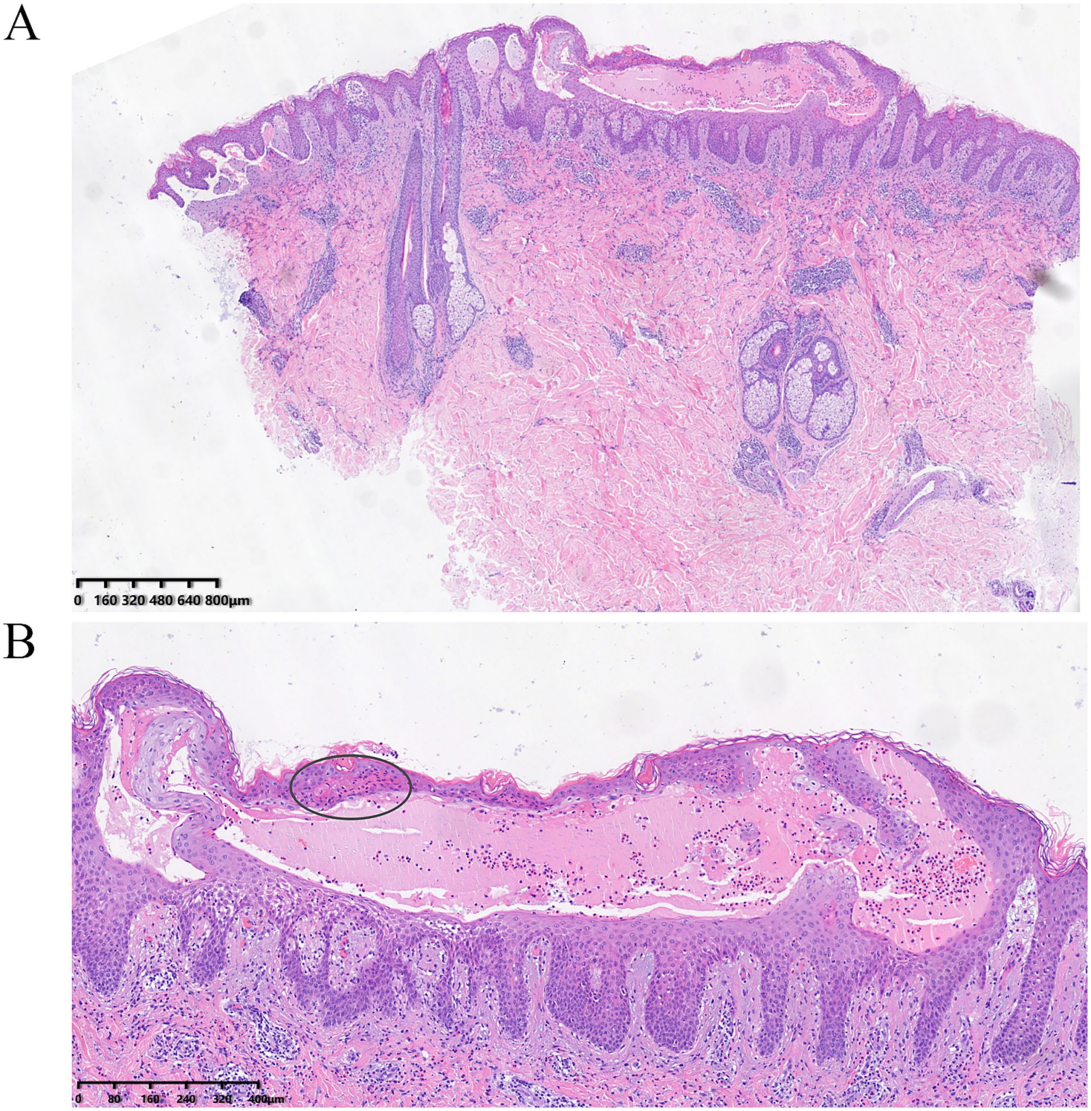
Dermatopathology Photomicrographs. **(A)** Low-power photomicrograph of the skin biopsy. **(B)** High-power photomicrograph showing necrotic keratinocytes and basal cell liquefaction degeneration. Necrotic keratinocytes are indicated by black circle. Scale bars are shown in the images.

Laboratory examinations showed an elevated absolute eosinophil count of 1.29 × 10^9^/L (reference range: 0.02–0.52 × 10^9^/L), an eosinophil percentage of 16.8% (reference range: 0.4–8%), and the immunoglobulin E (IgE) level was 3,557 IU/mL. Serological testing showed positivity for herpes simplex virus type I/II IgG and negativity for herpes simplex virus type I/II IgM. Mycoplasma pneumoniae antibody testing was negative. Results for the immunoglobulins and complement, antinuclear antibodies, anti-epidermal keratinocyte desmosome and basement membrane antibodies, ESR, CRP, biochemistry, coagulation, and infection screening were all within normal limits. The patient also had no history of other recent medication exposure apart from the treatment prescribed for urticaria, and the above negative infectious and autoimmune work-up made common infectious, autoimmune bullous, and alternative drug-related causes less likely. Skin biopsy pathology obtained from the left upper limb showed focal sheet-like necrotic keratinocytes in the epidermis, liquefaction degeneration of the basal cells, and intraepidermal and subepidermal blister formation containing cystic fluid and a large amount of eosinophilic infiltration (Figure 2). Sheet-like eosinophilic and lymphocytic infiltration was observed around the small vessels in the dermis. Based on the history of omalizumab treatment, the typical lesions and the histopathological findings, the diagnosis of “omalizumab-induced bullous erythema multiforme” was made. The causal relationship between omalizumab and bullous erythema multiforme was assessed using the Naranjo Adverse Drug Reaction Probability Scale. The total score was 6, indicating a probable adverse drug reaction. The detailed scoring table is provided in the Supplementary Table S1.

### 2.2 Treatment

**Figure 3:**
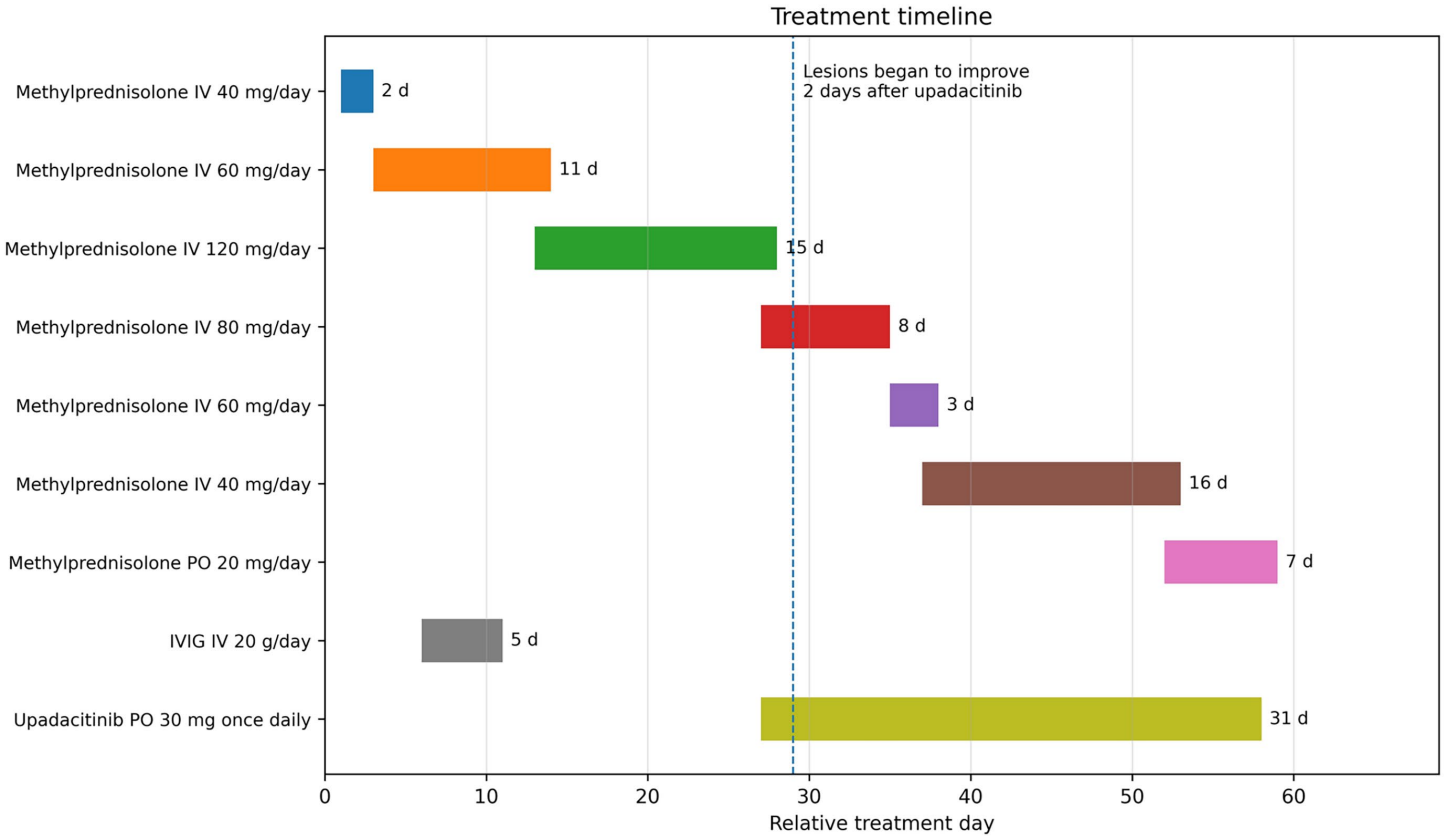
The treatment timeline during the patient’s hospitalization.

**Figure 4:**
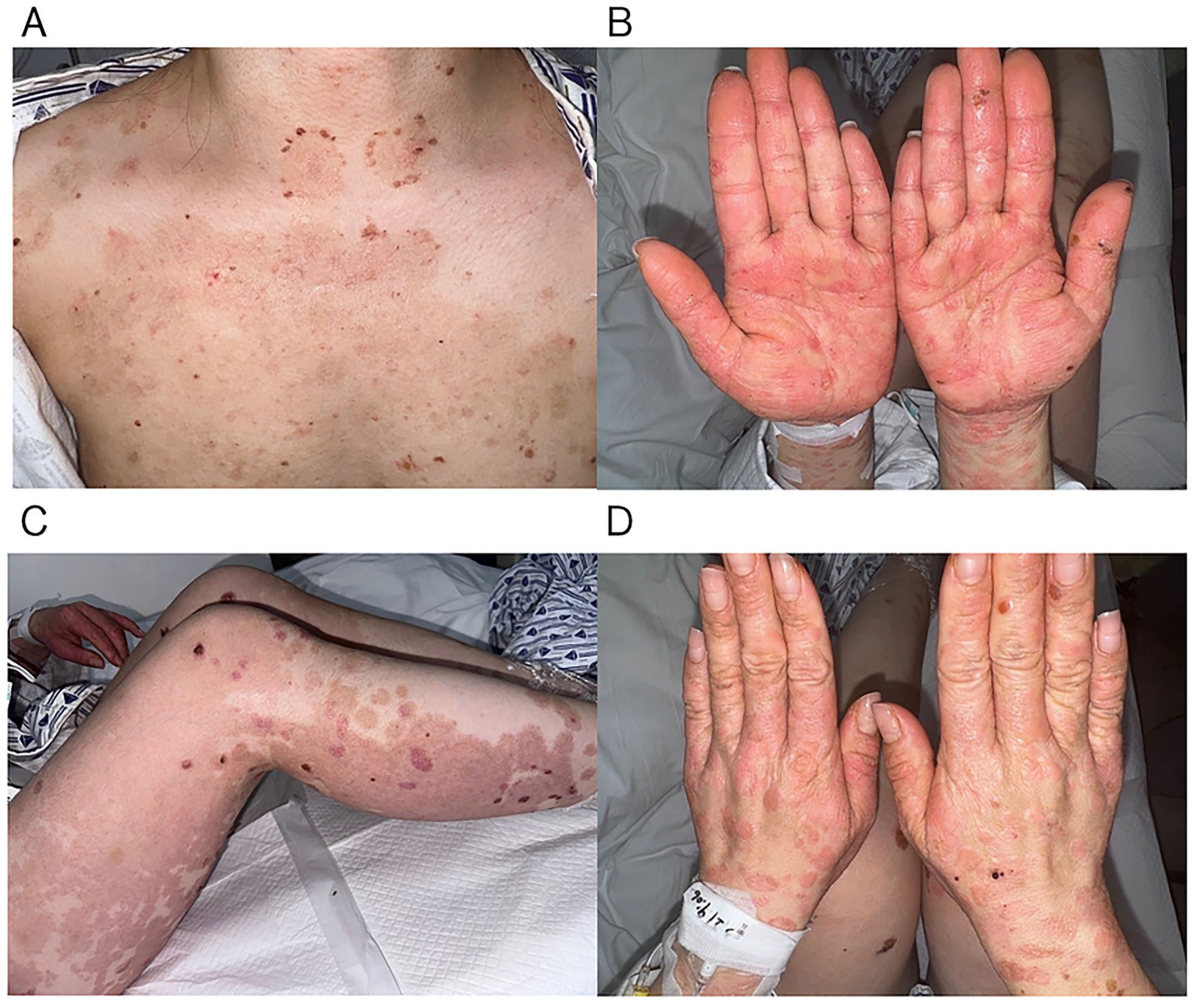
Clinical images taken on day 52 after hospital admission. **(A)** Residual erythematous macules and post-inflammatory hyperpigmentation on the trunk. **(B)** Residual erythema and desquamation on the palms. **(C)** Residual erythematous lesions and post-inflammatory hyperpigmentation on the lower limb. **(D)** Residual lesions with post-inflammatory hyperpigmentation on the dorsal hands.

Initial treatment consisted of intravenous methylprednisolone at 40 mg/d combined with intravenous immunoglobulin (IVIG) at 20 g/d. However, the skin lesions continued to progress, and the peripheral blood eosinophil count increased further. Subsequently, the dose of methylprednisolone was increased to 60 mg/d, but new vesicles continued to appear on the limbs. Consequently, the dose of methylprednisolone was increased to 120 mg/d, combined with oral upadacitinib at 30 mg/d. Following this, the skin lesions began to improve after 2 days, the blisters dried and crusted, and no new vesicles or bullae appeared. The treatment timeline during the patient’s hospitalization is shown in Figure 3. During systemic corticosteroid treatment, potassium chloride 0.5 g twice daily, omeprazole 20 mg once daily, and calcium carbonate 600 mg once daily were administered as supportive prophylactic medications. Complete blood counts, lipid profile, blood glucose, and electrolyte levels were monitored regularly and remained within normal ranges. No treatment emergent adverse effects related to methylprednisolone or upadacitinib were observed during hospitalization. At discharge, the patient’s condition was stable, and the corticosteroid dose was gradually tapered thereafter (Figure 4). At the 3 month follow-up after discharge, the erythematous lesions had almost completely resolved, leaving only residual post-inflammatory hyperpigmentation, and upadacitinib was reduced to 15 mg once daily. At present, the patient remains clinically stable and is taking one tablet of upadacitinib every other day.

## 3 Discussion

The estimated incidence of drug-induced BEM is less than 10% (6). As a recombinant humanized monoclonal antibody, Omalizumab blocks IgE-mediated inflammatory pathways and is clinically used primarily for chronic spontaneous urticaria and asthma (7). Severe cutaneous bullous adverse reactions induced by it are extremely rare. In this case, the histopathological examination confirmed drug-induced BEM. The patient developed typical multiple raised, edematous erythematous papules and plaques and bullae 17 days after her first dose of omalizumab, and infections or other medication histories were excluded. Therefore, omalizumab was identified as the causative agent for this episode of BEM. A previous pharmacovigilance case report described omalizumab-induced erythema multiforme in a patient with chronic spontaneous urticaria (8). However, detailed reports of biopsy supported bullous erythema multiforme after omalizumab exposure remain extremely limited. To our knowledge, the present case is the first detailed report of omalizumab induced BEM controlled with high dose glucocorticoids in combination with upadacitinib.

The immunopathological mechanism of omalizumab induced BEM is not yet fully understood and may involve a complex dysregulation of immune homeostasis and antibody-triggered immune activation. A notable feature of this case is the elevated eosinophil infiltration observed in both peripheral blood and histopathology. Such marked eosinophilia is more suggestive of a drug reaction or bullous pemphigoid. This case should be differentiated from Stevens-Johnson syndrome (SJS) and autoimmune bullous diseases. Although oral mucosal involvement was present, the Nikolsky sign was negative, and there was no large scale epidermal necrolysis or confluent erythema, which is inconsistent with the clinical features of SJS (9). Erythema multiforme and SJS were once considered part of a disease spectrum but are now recognized as distinct entities. Conversely, SJS and toxic epidermal necrolysis (TEN) exist on a continuum (10). Additionally, laboratory tests were negative for anti-epidermal keratinocyte desmosome and basement membrane antibodies, ruling out pemphigus and bullous pemphigoid. The focal epidermal necrosis and massive eosinophilic infiltration observed in the skin biopsy further confirm that a drug-induced immune response is likely the initiating factor.

The patient’s treatment course reflects the refractory nature of such adverse reactions. Standard doses of glucocorticoids combined with intravenous immunoglobulin (IVIG) failed to rapidly suppress the condition, and skin lesions continued to progress during the initial treatment phase. Ultimately, the condition was controlled by increasing the glucocorticoid dose and combining it with the JAK inhibitor upadacitinib. As a selective JAK1 inhibitor, upadacitinib effectively blocks the signal transduction of various pro-inflammatory cytokines, including IL-4 and IL-5, playing a key role in inhibiting eosinophil activation and recruitment (11). Clinical evidence supporting JAK inhibition for erythema multiforme remains limited but is increasing. Murphy et al. reported a retrospective case series of four patients with persistent erythema multiforme treated with tofacitinib and/or upadacitinib, with mechanistic findings implicating interferon-*γ* and IL-15 signaling (12). Subsequent case reports also described successful control of persistent or recurrent erythema multiforme with upadacitinib (13, 14). These findings provide a biologically plausible rationale for the use of JAK inhibitors in refractory erythema multiforme.

This report has several limitations. First, observations from a single case cannot be generalized to broader patient populations. Second, because high-dose methylprednisolone and upadacitinib were initiated together, the independent therapeutic effect of upadacitinib cannot be determined. Further studies and accumulated case data from diverse populations are needed to clarify the risk factors, underlying mechanisms, and optimal management strategies for omalizumab-associated bullous adverse reactions.

Overall, this case highlights the therapeutic challenge of severe and refractory bullous drug eruptions associated with biologic agents. Early use of adequate-dose systemic glucocorticoids remains essential for rapid disease control. In patients who respond poorly to conventional therapy, adjunctive JAK inhibition may provide a biologically plausible and potentially effective therapeutic option.

## Data Availability

The raw data supporting the conclusions of this article will be made available by the authors, without undue reservation.
