# Supplementary material for: Case Report: Bullous erythema multiforme induced by omalizumab
Source: Front Med (Lausanne). 2026 Jul 17;13:1881122. doi: 10.3389/fmed.2026.1881122 (PMC13423651; doi:10.3389/fmed.2026.1881122)
Supplement: Supplementary file 1 [file Table_1.DOCX]

**Supplementary materials**

**Table S1. Naranjo Adverse Drug Reaction Probability Scale for omalizumab-induced bullous erythema multiforme**

| **No.** | **Naranjo assessment item** | **Answer in this case** | **Score** | **Rationale** |
| --- | --- | --- | --- | --- |
| 1 | Are there previous conclusive reports on this reaction? | Yes | +1 | A previous pharmacovigilance report described omalizumab-induced erythema multiforme in a patient with chronic spontaneous urticaria. |
| 2 | Did the adverse event appear after the suspected drug was administered? | Yes | +2 | The patient developed edematous erythema, vesicles, and bullae 17 days after the first subcutaneous injection of omalizumab. |
| 3 | Did the adverse reaction improve when the drug was discontinued or a specific antagonist was administered? | Do not know | 0 | Omalizumab was not re-administered; however, clinical improvement occurred after intensive systemic treatment, including high-dose methylprednisolone and upadacitinib. Therefore, an independent dechallenge effect could not be clearly determined. |
| 4 | Did the adverse reaction reappear when the drug was re-administered? | Do not know | 0 | Drug rechallenge was not performed for ethical and safety reasons. |
| 5 | Are there alternative causes that could have caused the reaction? | No | +2 | Infectious triggers and autoimmune bullous diseases were considered less likely based on the negative work-up, including negative herpes simplex virus IgM, negative Mycoplasma pneumoniae antibody testing, and negative autoimmune bullous disease-related antibody tests. No other recent medication exposure was identified apart from the treatment prescribed for urticaria. |
| 6 | Did the reaction reappear when placebo was given? | Do not know | 0 | Placebo challenge was not performed. |
| 7 | Was the drug detected in blood or other fluids at toxic concentrations? | Do not know | 0 | Serum omalizumab concentration was not measured, and a toxic concentration threshold is not clinically applicable in this setting. |
| 8 | Was the reaction more severe when the dose was increased or less severe when the dose was decreased? | Do not know | 0 | No dose escalation or dose reduction of omalizumab was performed. |
| 9 | Did the patient have a similar reaction to the same or similar drugs in any previous exposure? | No | 0 | The patient had no documented history of a similar reaction to omalizumab or related biologic agents. |
| 10 | Was the adverse event confirmed by objective evidence? | Yes | +1 | The diagnosis was supported by clinical targetoid and bullous lesions, mucosal involvement, and histopathological findings showing necrotic keratinocytes, basal cell liquefaction degeneration, blister formation, and prominent eosinophilic infiltration. |
|  | **Total score** |  | **6** | **A total score of 6 indicates a probable adverse drug reaction.** |
